# Supplementary material for: Topologically controlled circuits of human iPSC-derived neurons for electrophysiology recordings
Source: Lab Chip. 2022 Mar 7;22(7):1386–403. doi: 10.1039/d1lc01110c (PMC8963377; doi:10.1039/d1lc01110c)
Supplement: LC-022-D1LC01110C-s001 [file LC-022-D1LC01110C-s001.pdf]

Electronic Supplementary Material (ESI) for Lab on a Chip.
This journal is © The Royal Society of Chemistry 2022

Supplementary Information: Topologically controlled circuits of human iPSC-derived neurons for electrophysiology recordings

Sophie Girardin, Blandine Clément, Stephan J. Ihle, Sean Weaver, Jana B. Petr, José Mateus, Jens Duru, Magdalena Krubner, Csaba Forró, Tobias Ruff, Isabelle Fruh, Matthias Müller and János Vörös

1 iNeuron survival over time in PDMS microstructures

1.1 Survival rates

To estimate the survival rate, iNeurons were seeded into two sets of samples containing PDMS microstructures, as detailed in Section 2.5.1 of the main text. One of the sets of samples was supplemented with 1 µg/mL of laminin in the first week of culture (”Laminin" condition) while the other was not (”No laminin" condition). The samples were stained at DIV 0 and restained at DIV 11. The images were cropped into four individual images of nodes (N = 240 nodes per condition).

1.1.1 Image analysis

Fig. S1 shows representative images of a fluorescently labelled circuit of iNeurons. Such images were used to obtain the plots shown in Fig. 2. The number of live cells on DIV 0 and DIV 11 images were analysed according to the method presented in section 2.5.1 of the main paper. The number of dead cells at DIV 0 was estimated by processing the red channel of the image of each node. The red channel image was smoothed using a mean filter with a radius of one pixel. Default thresholding was then used to obtain a binary mask[1], followed by the built-in Fiji Watershed algorithm to separate neighboring particles. Finally, the number of particles was counted for each mask.

Fig. S2a and b show a step-by-step example of the automatized counting of live and dead cells for an example node for both the "no laminin" and "laminin" conditions. Fig. S2c compares the numbers obtained from the images shown in (a) and (b) with the manual hand count. Automatized counting is within 15% of the manual count. Fig. S2d shows the manual count of the live cells at DIV 11. By DIV 11, dead cells were not uniformly stained and some of them had separated into several pieces, making the use of segmentation unreliable to count the number of dead cells.

1.1.2 Survival rate at DIV 0

Survival rates at DIV 0 were calculated for both sets of samples. The survival rate of node i at DIV 0 ($r_{0,i}$) was calculated for each node as:

$$r_{0,i} = \frac{n_{\text{live},0,i}}{(n_{\text{live},0,i} + n_{\text{dead},0,i})}$$

with $n_{live,0,i}$ the number of live cells and $n_{dead,0,i}$ the number of dead cells at DIV 0 for node i.

The resulting survival rates at DIV 0 for both sets of samples can be seen in Fig. S3. Importantly, laminin was added to the medium of the second set of samples at the same time as the CMFDA/ethidium homodimer-1 stains, 1 h after seeding. In the samples where no laminin was added, the average survival rate was 73%, whereas in the samples where laminin was added, the survival rate at DIV 0 was 67%. The null hypothesis of the statistical test that we used to compare both sets of samples was that there was no difference in the distribution of the percentage of live cells between the two samples at DIV 0. The survival rate was significantly higher in the samples without laminin vs. the samples where laminin was added. As laminin is not expected to have an instant effect on the samples, the differences observed in the initial survival between the two sets of samples is not attributable to the addition of laminin, but likely due to stochastic variations in the number and survival of cells contained in the volume pipetted onto the sample during the initial cell seeding.

1.1.3 Absolute survival rate at DIV 11

Fig. 2c presents the survival rate at DIV 11 relative to the number of cells alive at DIV 0. Based on the number of live and dead cells estimated from the DIV 0 and DIV 11 images, it is also possible to calculate the absolute survival rate at DIV 11, *i.e.* the survival rate relative to the total number of cells seeded at DIV 0. The absolute survival rate of node i at DIV 11 ($r_{11,i}$) can be calculated for each node as:

$$r_{abs,11,i} = \frac{n_{\text{live},11,i}}{(n_{\text{live},0,i} + n_{\text{dead},0,i})}$$

with $n_{\text{live},11,i}$ the number of live cells at DIV 11 for node i.

Here we assume that the total number of cells per node did not vary between DIV 0 and DIV 11 and used the total cell count from DIV 0 for each respective node to calculate the absolute survival rate. This was possible because the same circuits were stained at DIV 0 and restained at DIV 11. Estimating the number of dead cells from DIV 11 images was impossible, as by that time, many of the dead cells had degraded into several pieces and were overlapping, making it difficult to get a reliable count of dead cells from the red channel images (see Fig. S2d).

The resulting absolute survival for both the ”laminin" and the ”no laminin" conditions can be seen on Fig. S4. For the ”no laminin" samples, the average survival is of 0.4%, while it is of 2.9% for the ”laminin" samples, a significant difference. In this case, the difference in survival at DIV 11 can be attributed to the addition of laminin in the medium.

1.2 Area measurement of green- and red-stained structures

Fig. S5 shows representative images of fluorescent labelled circuits of iNeurons at DIV 4 and 7. Such images are the ones that were used to obtain the plots shown on Fig. 3. Each image of a full circuit was cropped into its four nodes. The area of the node occupied by green- and red-stained structures at the different DIVs was measured according to the method presented in section 2.5.2 of the main text. Fig. S6 shows examples of the steps used to obtain the binary masks from which the area occupied by live and dead cells in the node was calculated. The percentage area was obtained by dividing the white area by the area of the node ($2.27\text{x}10^{-}2$ mm^2).

2 Open cultures of iNeurons

To investigate survival in the absence of PDMS microstructures and determine if PDMS is the leading cause of the low survival rate of iNeurons, iNeurons were plated on bare PDL-coated glass at a high density (300 k cells/cm^2) and the change in area occupied by live and dead structures over night was investigated.

2.1 Substrate preparation and staining

A glass bottom 48-well plate (P48G-1.5-6-F, Mattek) was used as a substrate. It was plasma cleaned for 2 min and coated with 0.1 mg/mL PDL in PBS for 45 min before being rinsed 3 times with PBS and left in ultrapure water. iNeurons were seeded at a density of 300 k cells/cm^2 on 18 of the wells of the glass bottom well plates. Two different conditions were tested: culturing the samples with regular medium (9 wells) and with medium containing 1 µg/mL of laminin (9 wells). At DIV 0, 1, 2, and 3, two wells of each condition were stained with CMFDA and ethidium homodimer-1 and 15 to 25 fields of view were taken for each well. At DIV 10, one well of each condition was stained with the live/dead and Hoechst stains and 16 fields of view were imaged. The other 16 wells were restained to investigate the effect of early staining on cell survival.

The area occupied by green- and red-stained structures were calculated using image segmentation, as detailed in Section 2.5.2 of the main text. To calculate the percentage of the area occupied by green- or red-stained structures, the measured area (in µm^2) was divided by the total area of a field of view. Fig. S7 shows examples of the steps used to obtain the binary masks from which the area occupied by live and dead cells was calculated. Due to the microscopy settings used, fields of view from DIV 0 were smaller than images from the other DIVs (424 μm vs 626 μm of side). This was taken into account by calculating the percentage of the field of view occupied by live- or red-stained structures.

2.2 Change in the area occupied by live and dead iNeurons over time

Fig. S8a and b show representative images of stained live and dead cells in open cultures at DIV 0 and DIV 10, both in regular medium and in 1 µg/mL laminin-supplemented medium. Fig. S9 shows representative images of fluorescently labelled iNeurons at DIV 1, 2 and 3. From DIV 2, live iNeurons tended to cluster and overlap, making it difficult to reliably count the number of cells per field of view. For that reason, the area of the field of view occupied by green- or red-stained structures was used as a proxy for investigating the change over time of live and dead neurons. The area occupied by live and dead cells was hypothesized to correlate with the number of live and dead cells.

Fig. S8c and d show the area occupied by green- and red-stained structures over DIV 0, 1, 2, 3 and 10 for the ”no laminin" and the "laminin" samples. A statistical test was run to test the difference in the distribution of both sets of

samples. Because laminin was added at the same time as the stains on DIV 0, which is not expected to have an immediate effect on survival, the null hypothesis for the statistical test on DIV 0 was: there is no difference in the distribution of the percentage of live cells in the two initial sets of wells, consisting in thawed iNeurons. On DIV 1 to 10, laminin had been added to the samples for more than a day. The null hypothesis for these experiments was therefore: there is no difference in the distribution of the percentage of live cells in the samples supplemented with 1 μg/mL of laminin for a week (“Laminin”) vs. in the samples that were not supplemented with laminin (“No laminin”).

The area occupied by live iNeurons decreases by a factor of 4 in the first two days of cultures, before gradually decreasing until DIV 10 (Fig. S8c). Adding laminin to the medium does not have a measurable impact on the area occupied by live cells at DIV 10. There is a statistically significant difference in the area occupied by live cells at DIV 0 between the "no laminin" and "laminin" conditions. This difference is likely due to stochastic differences in the number of cells initially pipetted onto the substrates.

Based on the red-stained area measurements, the number of dead cells is fairly constant over the first three days, before slowly increasing between DIV 3 and DIV 10 (Fig. S8d). The area occupied by dead cells is on average higher in the "laminin" than in the "no laminin" condition at DIV 1, 3 and 10, with a statistically significant difference at DIV 1 and 3. This could be explained by the fact that laminin tends to make the surface of the substrate slightly more cell-adhesive than bare PDL, leading to more dead cells adhering to it rather than getting washed away during medium changes. Overall, even in an open culture surface at a rather high cell density, many of the iNeurons die over time, especially in the first few days of culture.

2.3 Effect of CMFDA/Ethidium homodimer-1 staining in early days of culture

We observed that staining cultures at early DIV has an adverse effect on the cell survival at later DIVs. We could quantify that effect by restaining at DIV 10 the wells of the 48-well plate used to obtain the plots shown on Fig. S8. Results of the area occupied by live structures on restained and non-restained wells can bee seen on Fig. S10. Compared to wells that were stained for the first time at DIV 10, restaining wells that had already been stained at DIV 0, 1, 2 or 3 resulted in 1 to 1.5 times lower area occupied by live cells. Early stains thus seem to have an adverse effect on survival and should be avoided. They can be replaced with genetically expressed fluorescent proteins. However, if using a typical cytosolic fluorescent proteins such as GFP, dead cells might also have expressed the fluorescent protein before dying, leading to unclean fluorescent images that usually cannot be automatically segmented and analyzed.

3 Protocol optimization

3.1 PDMS treatment

To determine if part of the cell death could be attributed to poorly treated PDMS, the effect of different PDMS cleaning methods prior to substrate making was assessed. The cleaning methods tested were autoclaving, solvent extraction and ethanol (A15, Thommen-Furler AG). Autoclaving consisted in placing the PDMS micostructures in an autoclave (Varioklav 75T, Sterico) and heating them at 121°C and 110 kPa for 20 min, followed by a 20 min drying cycle at a temperature of 81°C to 91°C. Solvent extraction was performed according to the extraction protocol reported by Millet *et al.*[2]. Ethanol cleaning consisted in immersing the PDMS membrane in ethanol for approximately 16 h, followed by 24 h of drying in an oven at 60°C.

Fig. S11 shows the distribution of the number of nodes with at least one live iNeuron for the different PDMS cleaning methods. It seems like the different cleaning methods had either little effect (autoclaving) or an adverse effect on the number of full circuits (solvent extraction and ethanol), so none of these PDMS treatments were kept in the substrate preparation protocol.

3.2 Macrophage co-culture

Because of the poor survival rate, dead iNeurons accumulate in the nodes of the PDMS circuits and might have an adverse effect on the remaining live iNeurons in the node. To test for this, iPSC-derived macrophages were added to the cultures on DIV 4. iPSC-derived macrophages were obtained following the protocol described by Giorgetti *et al.*[3] and kindly provided by Novartis as a suspension.

Upon reception, macrophages were centrifuged for 5 min at 1000 rpm, resuspended in macrophage medium and plated into a non-coated 6-well plate (92006, TPP). Macrophage medium consisted in RPMI 1640 GlutaMax (61870-036, ThermoFisher) with 10% heat inactivated FBS (A156-152, ThermoFisher), 1% sodium pyruvate (11360-039, ThermoFisher),

1% pen-strep (15140, ThermoFisher), 50 μM of mercaptoethanol (31350-010, ThermoFisher) and 40 ng/mL of human M-CSF (216-MC-025, Biotechne). Macrophages were kept in incubator for 8 days. They were then detached using TrypLE (12604-013) and centrifuged at 500 rpm for 5 min. Macrophages were seeded on top of a DIV 4 culture of iNeurons in PDMS microstructures at a density of about 50 k cells/cm^2. Phase contrast images of the wells were taken every other day until DIV 20. At DIV 20, a live/dead and Hoechst stain was performed on them.

Fig. S12a shows an example of one node getting cleaned by the macrophages over time. Impressively, the macrophages seem to phagocytose all of the dead cells contained in the nodes. Macrophages can squeeze into the low part of the chamber (see DIV 8 image) and move around the chamber a lot.

Fig. S13 shows a comparison between macrophage-containing and no macrophage-containing cultures. In the presence of macrophages, live iNeurons tend to tightly cluster together and their axons are grouped into fairly straight bundle connecting all four nodes (see Fig. S12b and Fig. S13b). This is likely due to macrophages moving around the nodes and leading to a mechanical bundling of the axons along the most straight path connecting one node to the next. However, the presence of macrophages does not seem to lead to a big improvement on the number of full nodes. Adding macrophages lead to a more complex protocol, along with adding uncertainties of mixing several cell types whose *in vivo* interactions are not fully understood. For those reasons, co-culture of macrophages and iNeurons was not further explored.

3.3 Full circuits

Fig. S14 shows representative examples of circuits with four nodes containing at least one live iNeuron for all of the conditions listed in Fig. 4b. A stitched image of all of the 15 circuits from one PDMS membrane can be seen on Fig. S15. This was obtained with condition 7 (10μg/mL of laminin).

3.4 Statistical tests on protocol variations

The number of live iNeurons per circuit after 3 weeks (DIV 18 to 23) was counted for all of the conditions presented in Fig. 4b. The statistical significance of the difference of the number of cells per circuit was tested by running pairwise two-sided Mann Whitney U tests on each pair of conditions. Results can be seen in Table S1.

Table S 1 P-values obtained by running the Mann Whitney U test for all pairs of conditions listed in Fig. 4b. *: $p < 0.01$

Condition	2	3	4	5	6	7
1	0.10	0.02	2.14E-13 (*)	1.60E-18 (*)	1.63E-19 (*)	8.84E-15 (*)
2		0.21	5.96E-11 (*)	2.84E-17 (*)	3.09E-19 (*)	8.40E-15 (*)
3			1.12E-10 (*)	2.75E-17 (*)	3.88E-19 (*)	9.44E-15 (*)
4				2.01E-07 (*)	3.35E-17 (*)	9.79E-14 (*)
5					1.07E-11 (*)	1.07E-08 (*)
6						0.13

4 Directionality

The "stomach" design of the PDMS microstructure allows to guide axons in a clockwise directions in 90% of the cases. This is due to the shape of the chamber and to the properties of the axons. When an axon starts growing from a single soma (Fig S16a), it grows until it hits a wall, then tends to follow the wall. If it grows towards the clockwise direction, it can simply follow the output channel to the next node (Fig S16b and c). If the axon grows towards the counter-clockwise direction, it will in most cases be redirected to the side channel, either because the axon is already following the top wall and naturally continues in the side channel (Fig S16d) or because it cannot follow the sharp angle from the input channel (Fig S16e). In 10% of the cases, the axon manages to follow the sharp angle from the input channel (Fig S16f) and connects in the wrong direction.

All of these different possible cases were observed in circuits where few neurons survived. Examples for each case are shown in Fig S17, for both the 100-μm (top) and 170-μm (bottom) diameter node design. Fig S17f shows examples where several neurons were growing in the node.

5 Electrophysiology

5.1 Action potential waveforms

The action potential waveforms for all four electrodes of the circuit shown on Fig. 6 were extracted at DIV 36 and DIV 133. They are visible on Fig. S18.

5.2 Overlaid raster plot

Fig. S19 shows an overlay of the spikes detected from the four electrodes of a circuit during 60 s of spontaneous activity. The activity recordings come from the iNeuron circuit shown in Fig. 6 at DIV 21, 62, 90 and 133. Electrodes are color-coded as in Fig. 6. To fit 60 s of spikes in one plot, each second of recording is visualized as two stacks of 500 ms, for a total of 120 stacks for each DIV. Some sequence of spikes repeat themselves, such as the "red-blue-red" pattern visible at DIV 21 or the many "red-green" patterns visible at DIV 62. Some more complex patterns are also visible at DIV 62 and 90. DIV 133 presents fewer of these patterns. The presence of these patterns of spikes suggests a temporal dependence between some of the electrodes of the circuit, a possible sign of functional connectivity. This was further inspected in Fig. 8 and 9.

5.3 Average number of active electrode per circuit

The average number of active electrodes per circuit of four electrodes was plotted in Fig. S20a. This plot shows a very similar trend than Fig. 7a: the addition of laminin to the medium leads to a much higher percentage of active electrodes.

The average MFR over all electrodes is shown in Fig. S20b. Since more than half of the electrodes of the “no laminin” MEAs are inactive, the blue MFR is substantially reduced, giving a false impression that the presence of laminin increases the firing frequency of the circuits, when in fact it is not the case. The presence of laminin simply increases the number of circuits with live cells and hence the number of circuits firing, but it does not increase the firing rate of the live cells.

5.4 Axon growth on top of PDMS microstructures

MEA recordings were taken over 133 DIV. Around DIV 90, a CMFDA staining was performed to investigate axons growing on top of the PDMS microstructures. An example of the growth of axons for one of the samples is visible on Fig. S21. On the one hand, almost no live soma are visible on the top surface of the PDMS, consistent with the observations that neurons generally could not survive on top of the PDMS on the first day of seeding. On the other hand, a lot of dead cells are visible on the phase contrast image, seemingly interacting with the network of axons. Over time, axons seem to have grown from the live cells inside of the nodes onto the top surface of the PDMS. This is probably due to the presence of laminin and other proteins in the cell medium, which deposit over time on the surface of the PDMS. Growth of axons on top of the PDMS could be avoided by functionalizing the top surface with an anti-fouling molecule.

5.5 500 ms-long response to stimulation

Fig. S22 shows the full 500 ms post-stimulus response for the circuit shown in Fig. 9d. Bands corresponding to the temporally consistent response to the stimulus are mainly concentrated in the first 20 ms of recordings, so a zoom-in into the first 20 ms of the response can be seen in Fig. 9e.

References

1 C. A. Glasbey, CVGIP: Graphical Models and Image Processing, 1993, **55**, 532–537.

2 L. J. Millet, M. E. Stewart, J. V. Sweedler, R. G. Nuzzo and M. U. Gillette, Lab on a Chip, 2007, **7**, 987–994.

3 E. Giorgetti, M. Panesar, Y. Zhang, S. Joller, M. Ronco, M. Obrecht, C. Lambert, N. Accart, N. Beckmann, A. Doelemeyer, L. Perrot, I. Fruh, M. Mueller, E. Pierrel, S. Summermatter, M. Bidinosti, D. R. Shimshek, S. Brachat and M. Nash, Cell Reports, 2019, **29**, 1539–1554.e7.

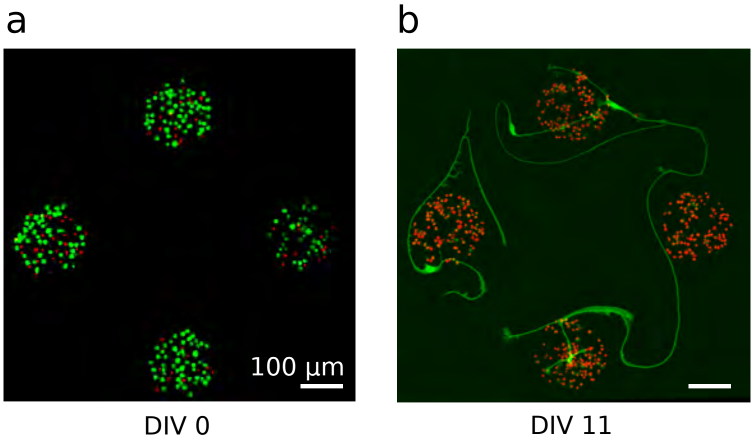

Figure S 1 Example of a circuit of fluorescently labelled iNeurons grown on a PDL-coated surface at DIV 0 (a) and at DIV 11 (b), without laminin supplemented to the culture medium. Both images come from the same circuit. At DIV 0, the green dye is CMFDA and at DIV 11, the green dye is Calcein AM (both staining live cells). For both, the red dye is ethidium homodimer-1, staining dead cells.

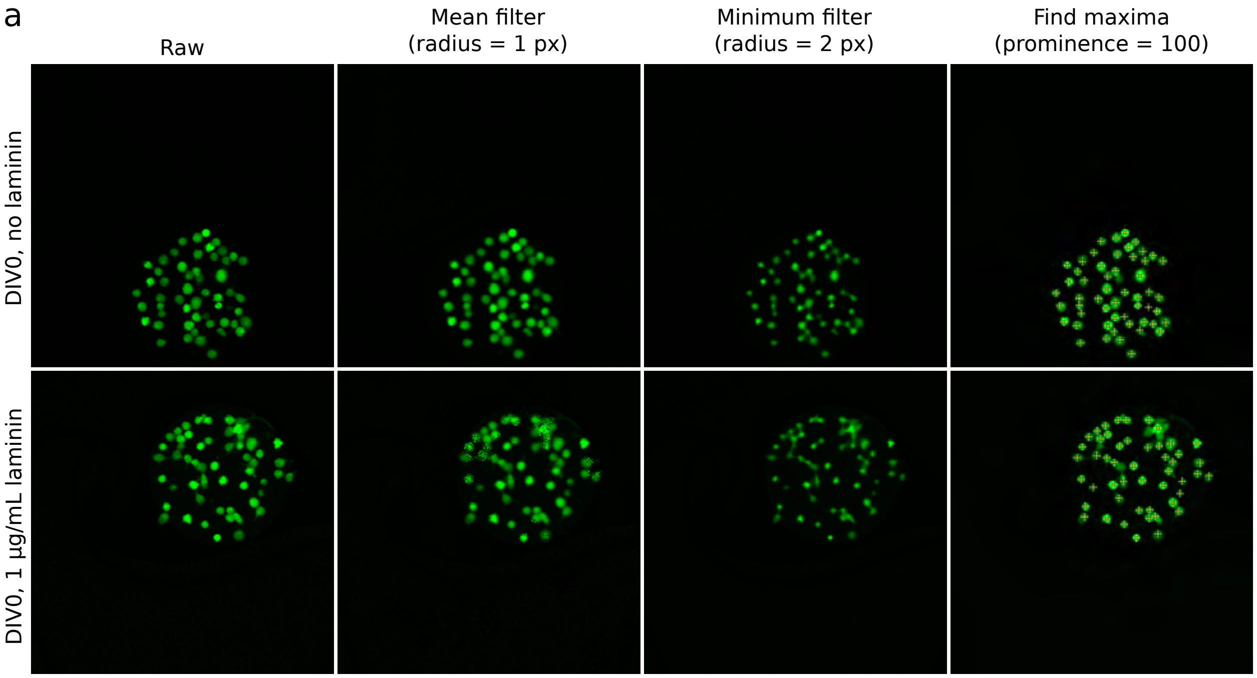

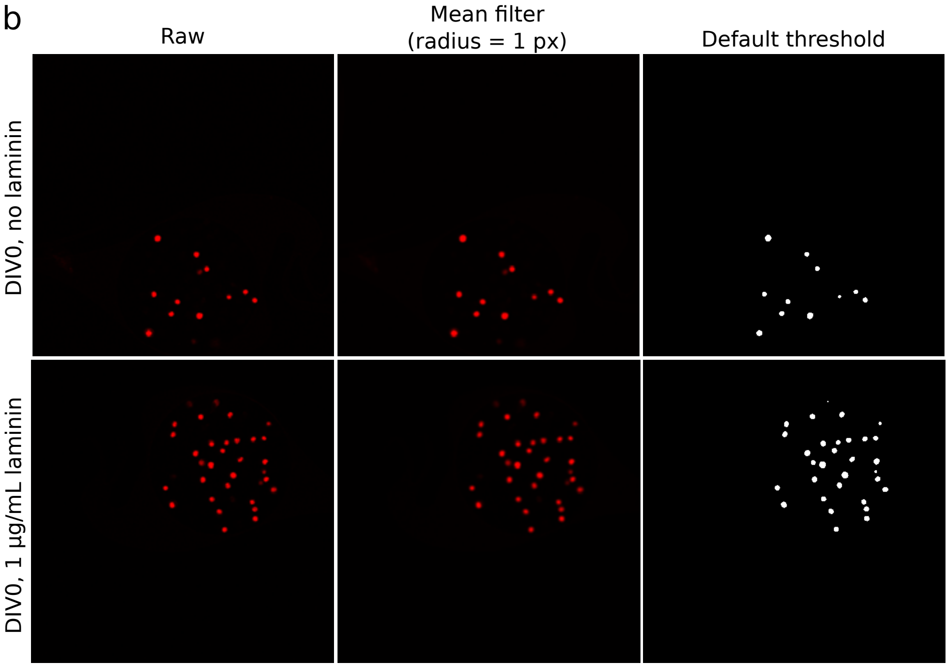

c

	DIV 0	Auto count	Hand count
Live	No lam	11	11
Live	Laminin	31	32
Dead	No lam	53	53
Dead	Laminin	49	56

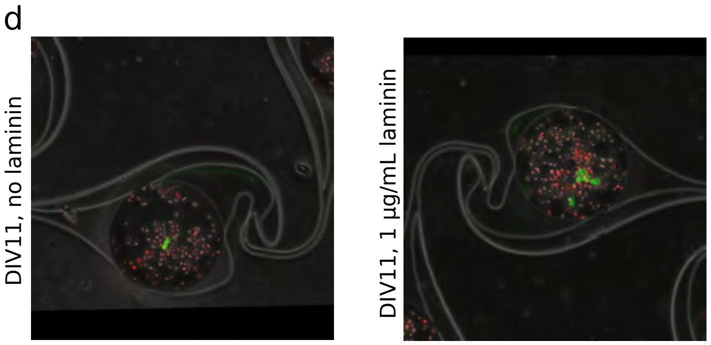

Figure S 2 Step-by-step image analysis to count the number of live and dead cells in DIV 0 and DIV 11 node images (for both the "no laminin" and the "laminin" conditions). (a) Analysis of nodes containing live cells. px: pixels. (b) Analysis of nodes containing dead cells. (c) Comparison between automated counting of the number of cells per node (using the pipelines shown in a and b) and a handcount of the number of cells per node. (d) At DIV 11, the live cells overlapped and the dead cells were not stained clearly enough to be automatically counted. For that reason, the number of live cell was manually counted, node by node.

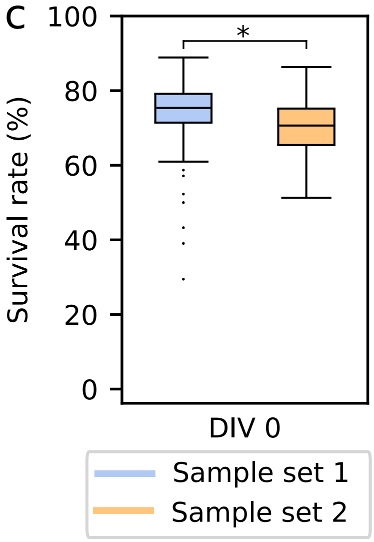

Figure S 3 Average survival rate per node for two different set of samples: the 2 samples which will then be cultured in regular medium (blue) and the 2 samples which will be cultured in medium supplemented with 1 µg/mL of laminin (orange), at DIV 0, before the addition of laminin

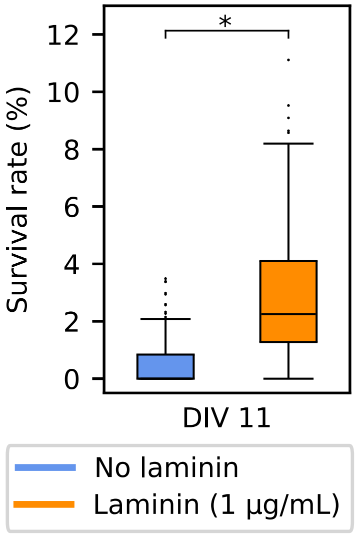

Figure S 4 Absolute survival rate per node at DIV 11 for samples cultured in regular medium (blue) and samples cultured in medium supplemented with 1 µg/mL of laminin (orange). The absolute survival rate was calculated based on on the initial number of cells seeded, including dead cells.

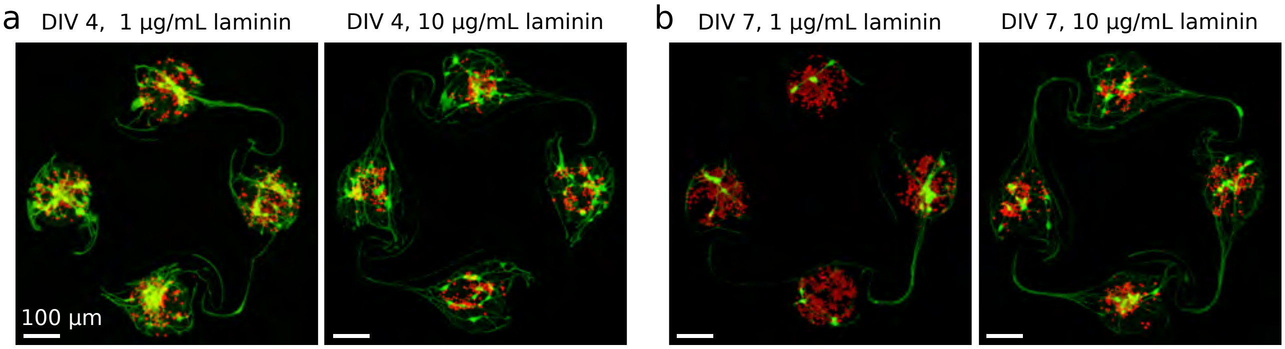

Figure S 5 Representative examples of fluorescently labelled iNeurons grown in PDMS microstructures at DIV 4 (a) and DIV 7 (b). Green: live cells, stained with CMFDA. Red: dead cells, stained with ethidium homodimer-1.

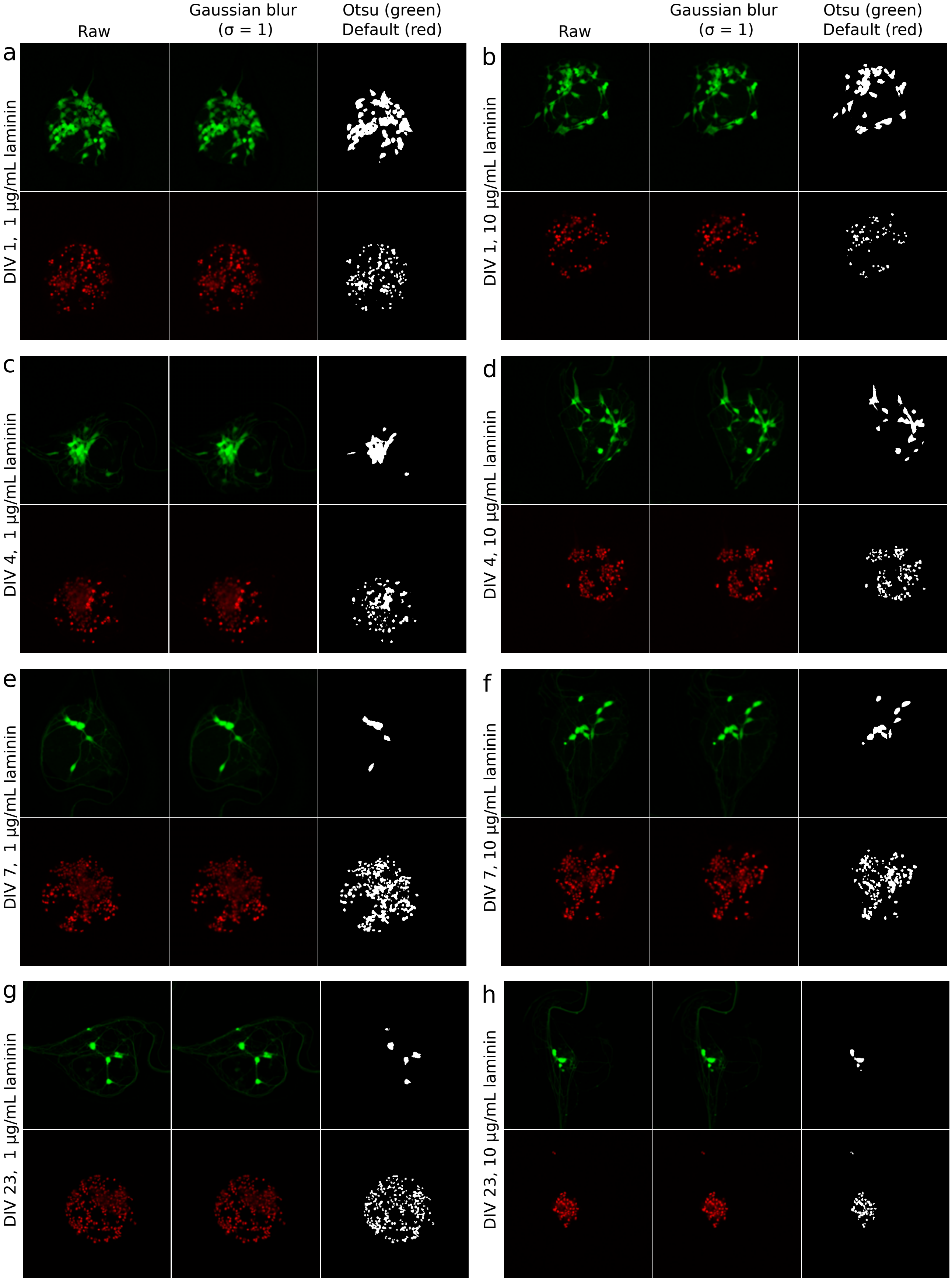

Figure S 6 Step-by-step image analysis to measure the area occupied by live and dead cells in images of nodes of PDMS microstructures. (a) DIV 1 - 1 μg/mL laminin (b) DIV 1 - 10 μg/mL laminin (c) DIV 4 - 1 μg/mL laminin (d) DIV 4 - 10 μg/mL laminin (e) DIV 7 - 1 μg/mL laminin (f) DIV 7 - 10 μg/mL laminin (g) DIV 23 - 1 μg/mL laminin (h) DIV 23 - 10 μg/mL laminin

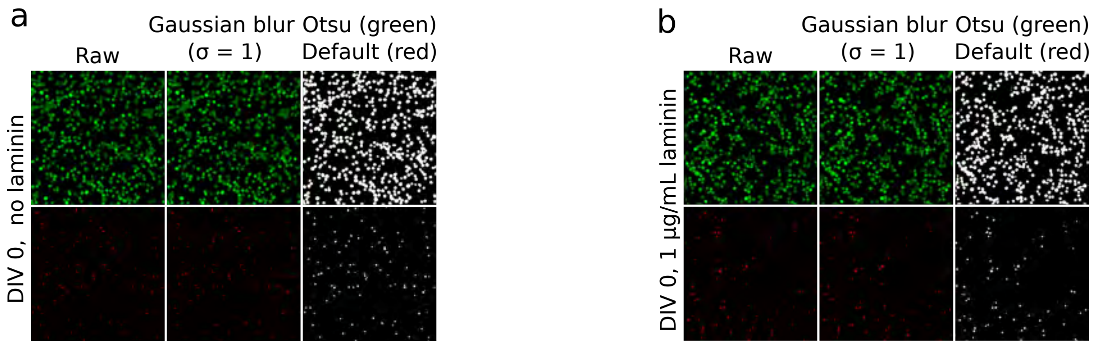

Figure S 7 [Continued on next page] Step-by-step image analysis to measure the area occupied by live and dead cells in images of open neuron cultures. (a) DIV 0 - no laminin (b) DIV 0 - laminin (c) DIV 1 - no laminin (d) DIV 1 - laminin (e) DIV 2 - no laminin (f) DIV 2 - laminin (g) DIV 3 - no laminin (h) DIV 3 - laminin (i) DIV 10 - no laminin (j) DIV 10 - laminin

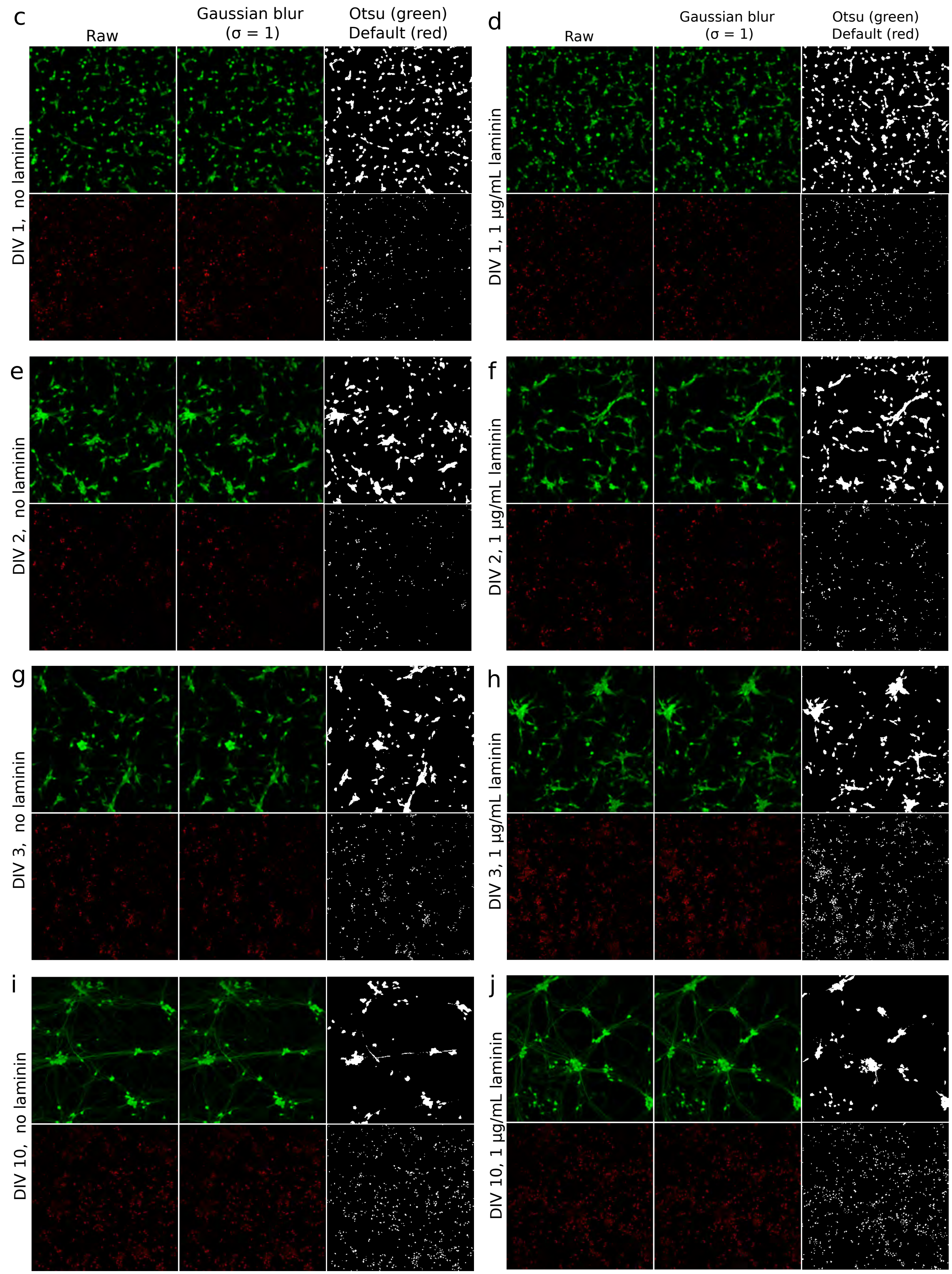

Figure S 7 See legend on previous page.

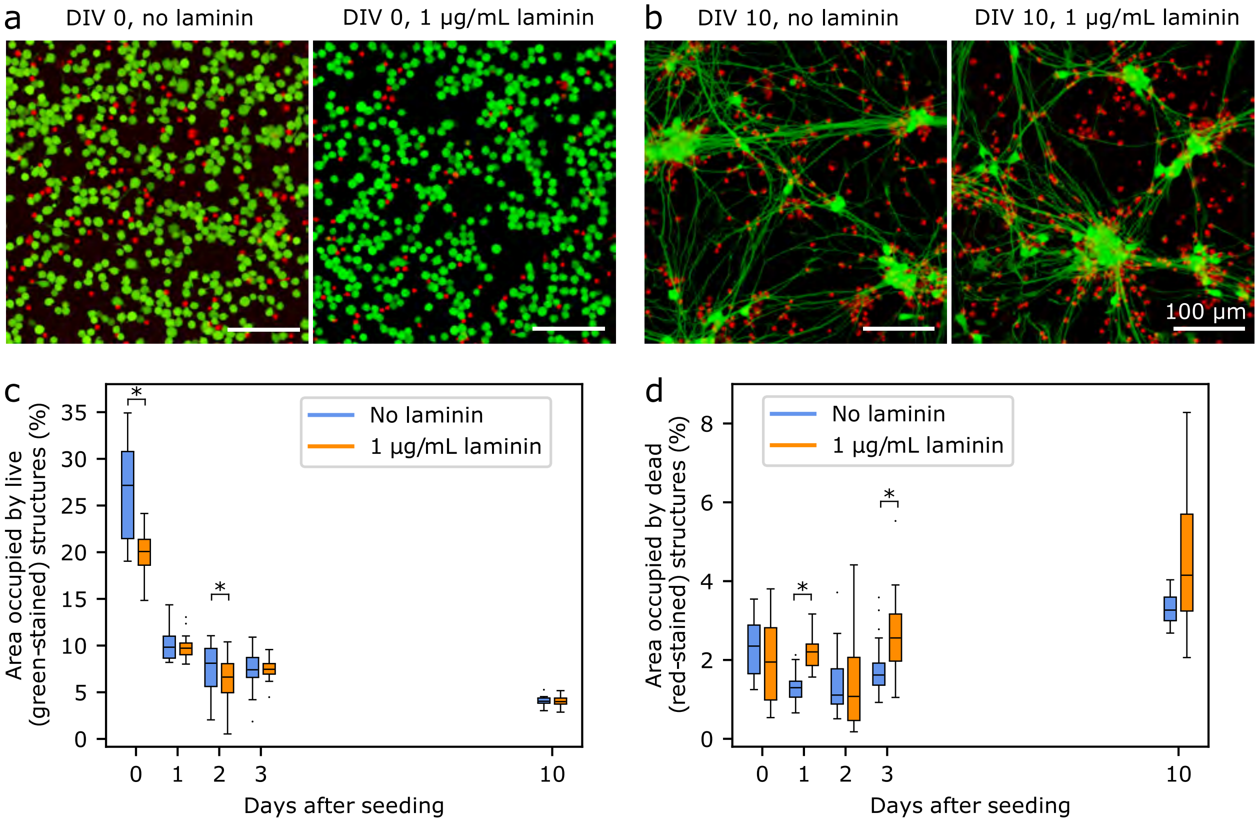

Figure S 8 Change over time of the area occupied by live and dead cells in open cultures of iNeurons. (a) Representative example of fluorescently labelled iNeurons grown on a PDL-coated surface at DIV 0, in regular medium (left) and in medium supplemented with 1 µg/mL of laminin (right). (b) Representative example of fluorescently labelled iNeurons grown on a PDL-coated surface at DIV 10 for the same two conditions. For both (a) and (b), live cells are labelled with the green stain Calcein AM and dead cells are labelled with the red stain ethidium homodimer-1. (c) Quantification of the change of average area occupied by green-stained structures (live cells) over time. (d) Quantification of the change of average area occupied by red-stained structures (dead cells) over time. For each box in (c) and (d), N = 13 to 50 fields of view, taken at random in open cultures of iNeurons. *: $p < 0.01$ (Mann Whitney U test).

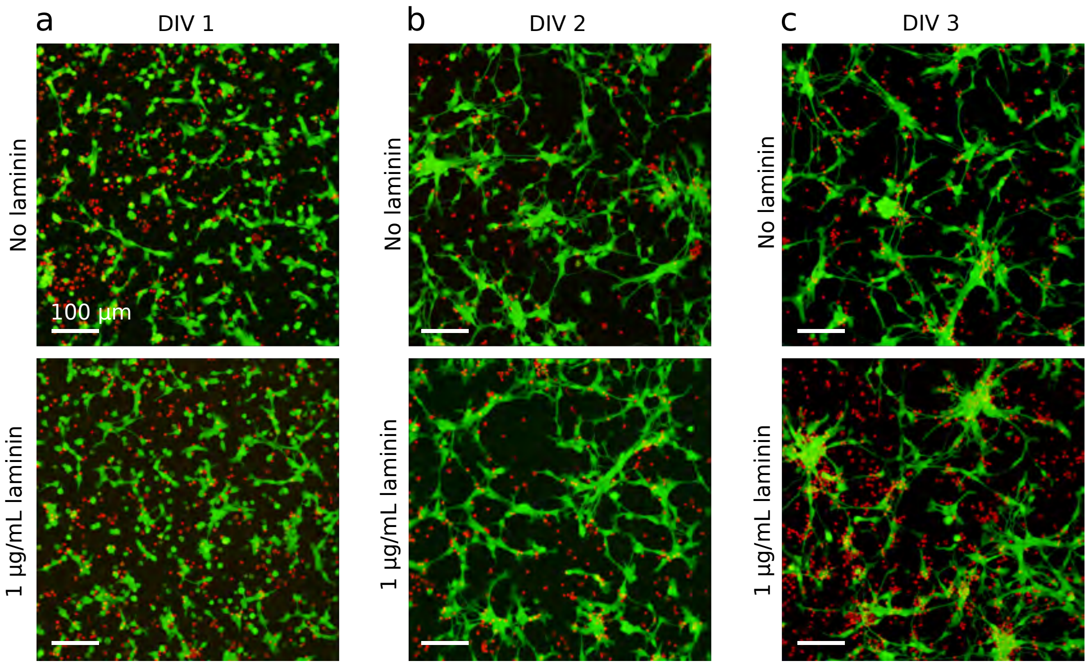

Figure S 9 Representative examples of fluorescently labelled iNeurons grown on a PDL-coated surface at DIV 1 (a), 2 (b) and 3 (c). Green: live cells, CMFDA. Red: dead cells, ethidium homodimer-1.

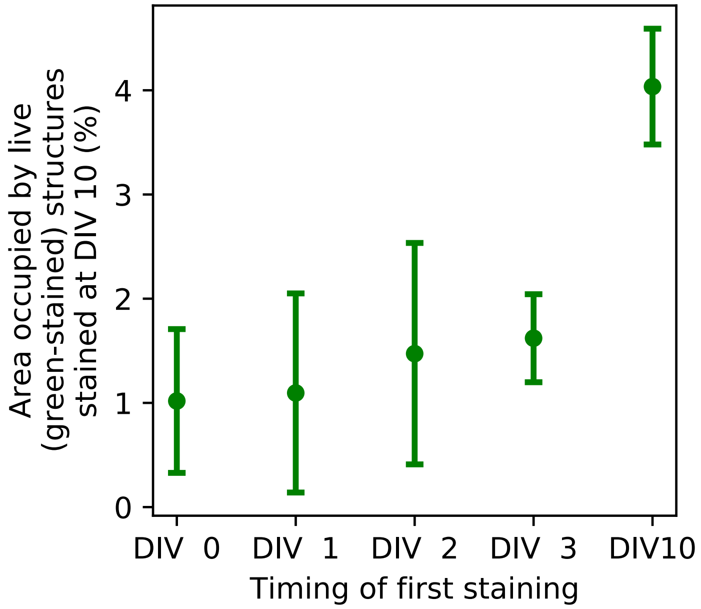

Figure S 10 Performing a first CMFDA/ethidium homodimer-1 staining in the early days of cell culture decreases the area occupied by green (live) structures at DIV 10, compared to a sample that was stained at DIV 10 for the first time. Staining at DIV 10 consisted in Calcein AM.

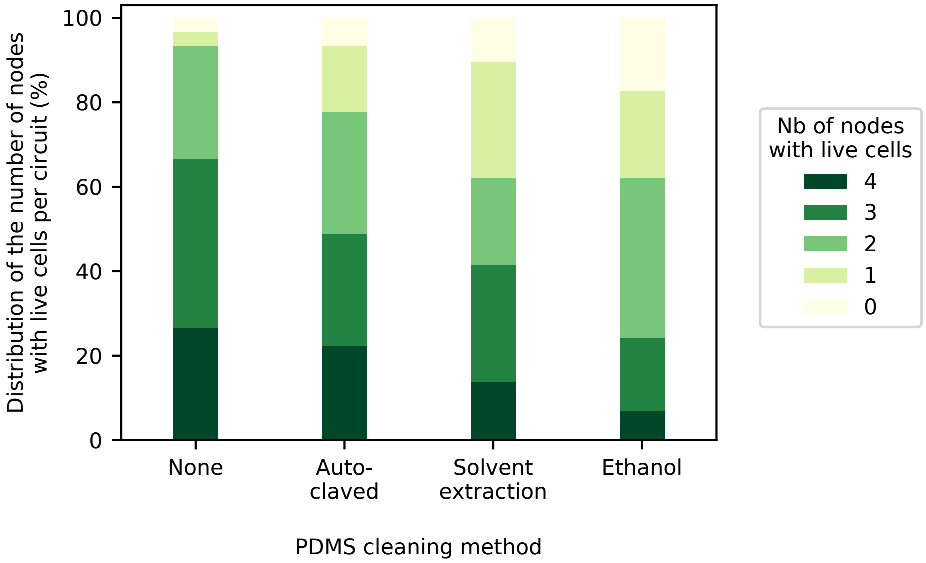

Figure S 11 Testing the effect of PDMS treatment on the survival of iNeurons in circuits.

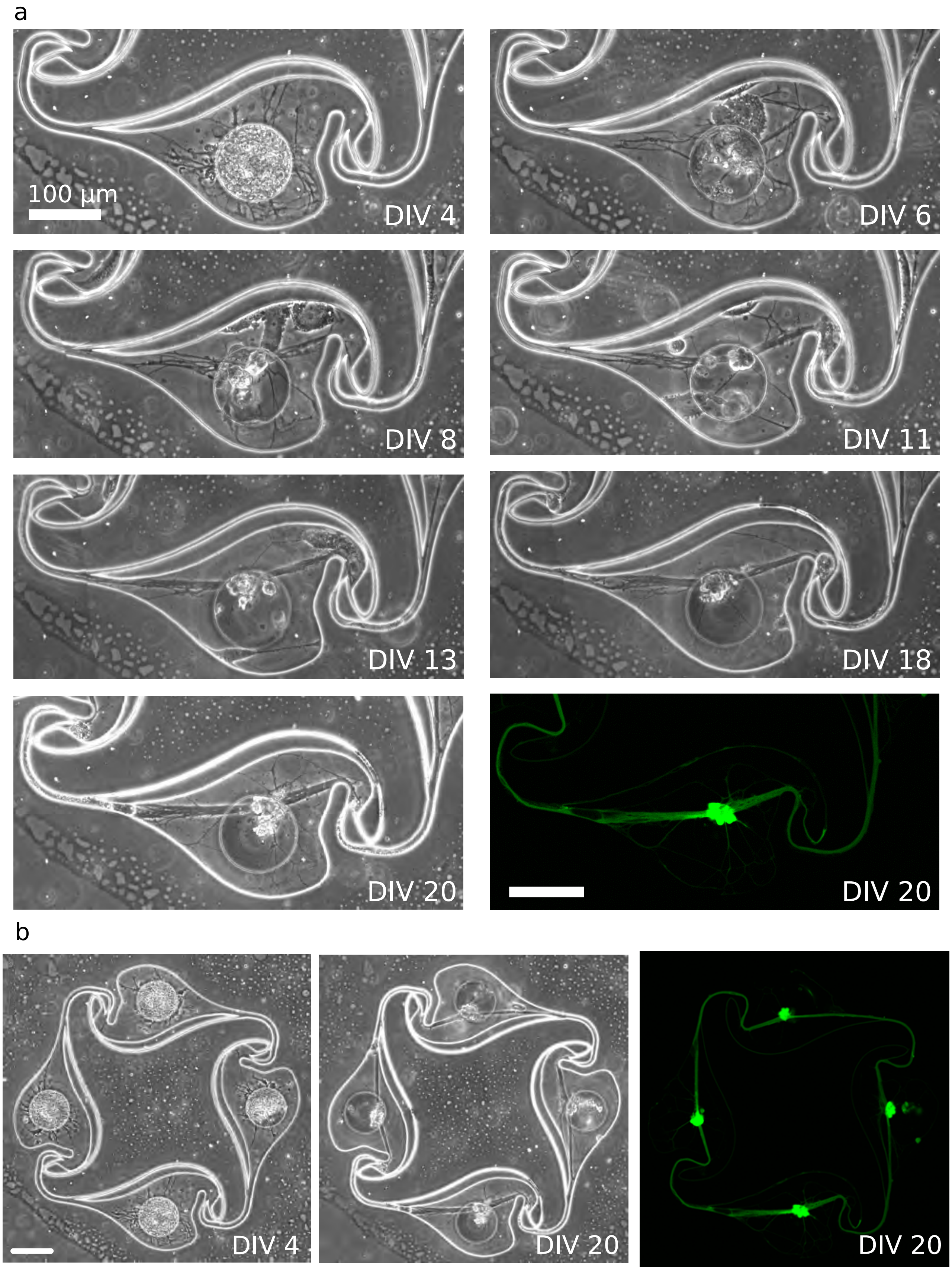

Figure S 12 Adding macrophages in nodes of iNeurons clears the dead cells. (a) Day-by-day evolution of a node initially filled with many dead iNeurons and where macrophages were added at DIV 4. Images are phase contrast, except for the bottom right one, which shows a Calcein AM fluorescent stain. (b) Full circuit shown in (a) for DIV 4 and DIV 20, with phase contrast and fluorescent Calcein AM stain. Scale bar: 100 μm.

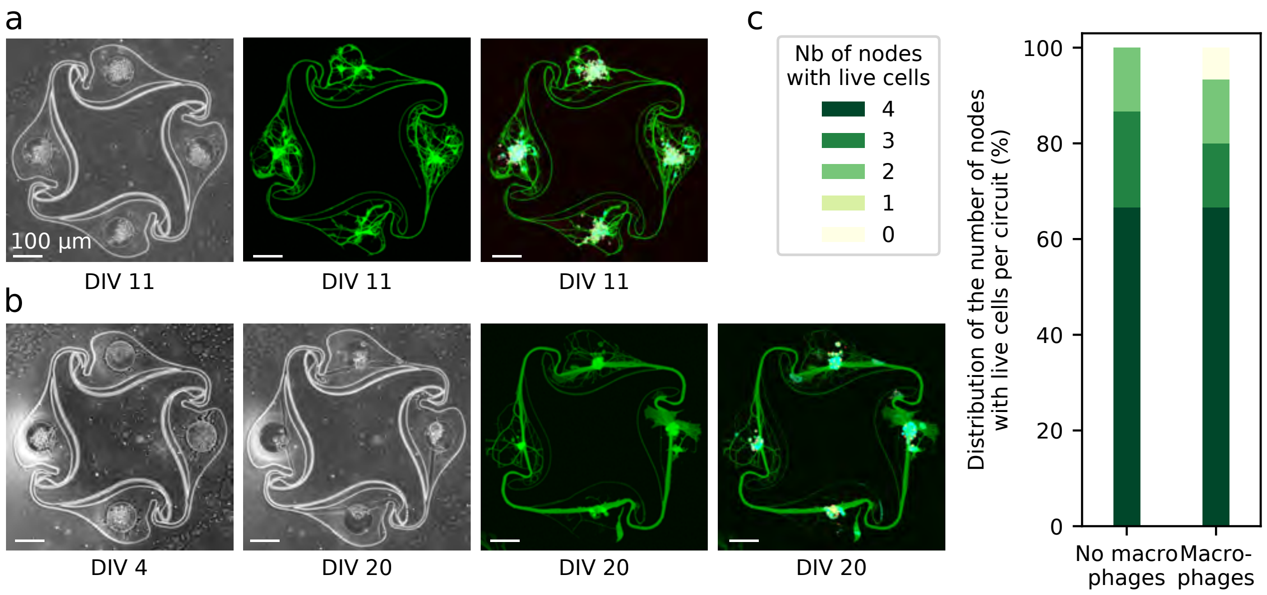

Figure S 13 Effect of the addition of macrophages on the number of full circuits. (a) Example circuit with iNeurons only, with no macrophages added to the circuit, at DIV 11. (b) Example circuit where macrophages were added to the circuit at DIV 4. Within a few days, the dead cells are cleared by the macrophages. For both (a) and (b): phase contrast pictures (left), Calcein AM stain (center) and overlay of Hoechst, Calcein AM and ethidium homodimer-1 stains (right). (c) Comparing the distribution of nodes with at least one live iNeuron with and without addition of macrophages at DIV 4 (N = 15).

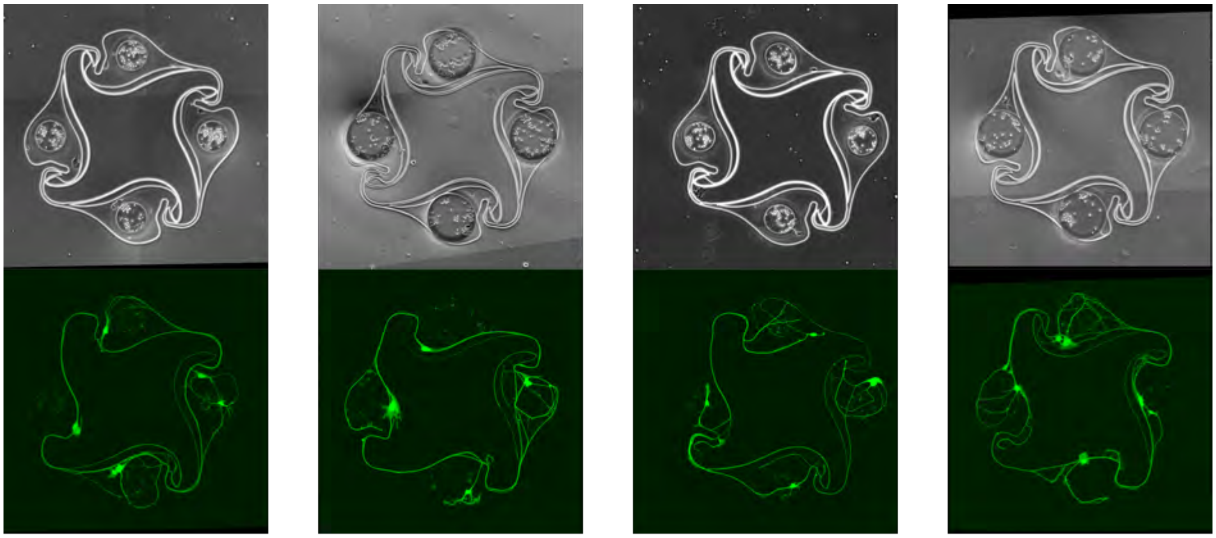

Condition 1
No laminin
30 k cells/cm^2
100 µm node

Condition 2
No laminin
30 k cells/cm^2
170 µm node

Condition 3
1 µg/mL of laminin
30 k cells/cm^2
100 µm node

Condition 4
1 µg/mL of laminin
30 k cells/cm^2
170 µm node

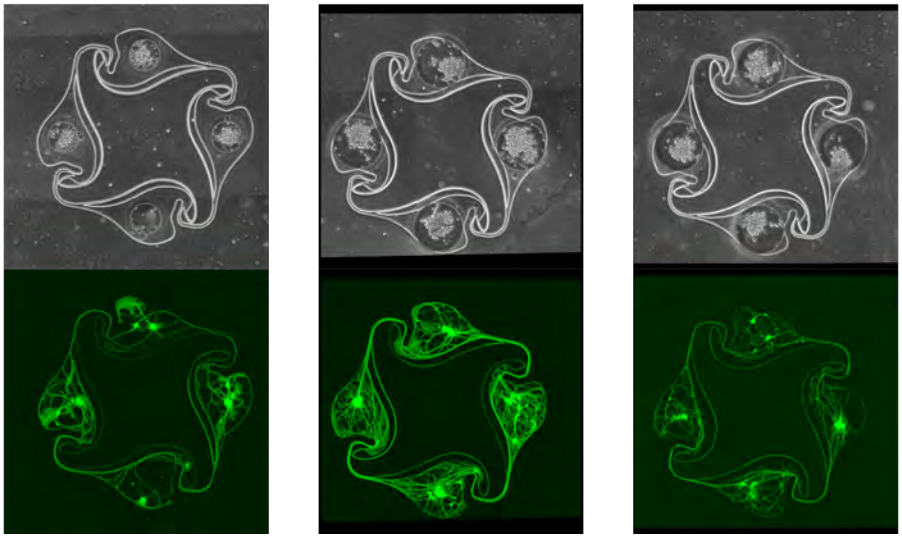

Condition 5
1 µg/mL of laminin
65 k cells/cm^2
100 µm node

Condition 6
1 µg/mL of laminin
65 k cells/cm^2
170 µm node

Condition 7
10 µg/mL of laminin
65 k cells/cm^2
170 µm node

Figure S 14 Representative examples of circuits with four nodes containing at least one live iNeuron each for the 7 conditions presented in Fig. 4b.

a
b
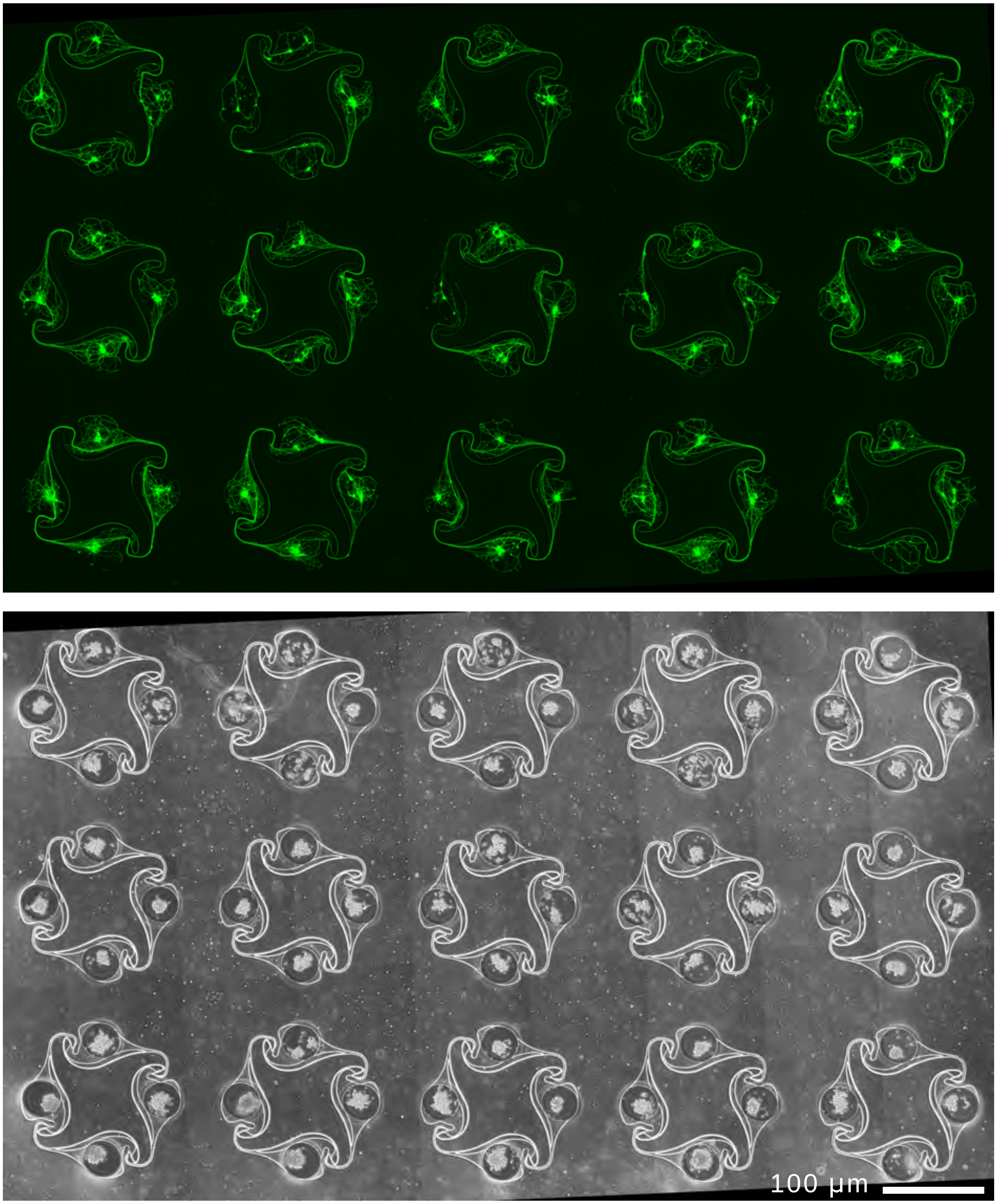

Figure S 15 Stitched image of the 15 circuits of a PDMS membrane. Top: Calcein AM; bottom: phase contrast. Circuits were seeded at a density of 65k cells/cm^2 and 10 μg/mL of laminin was added to the culture medium during the first week of culture (condition 7 in Fig. S14).

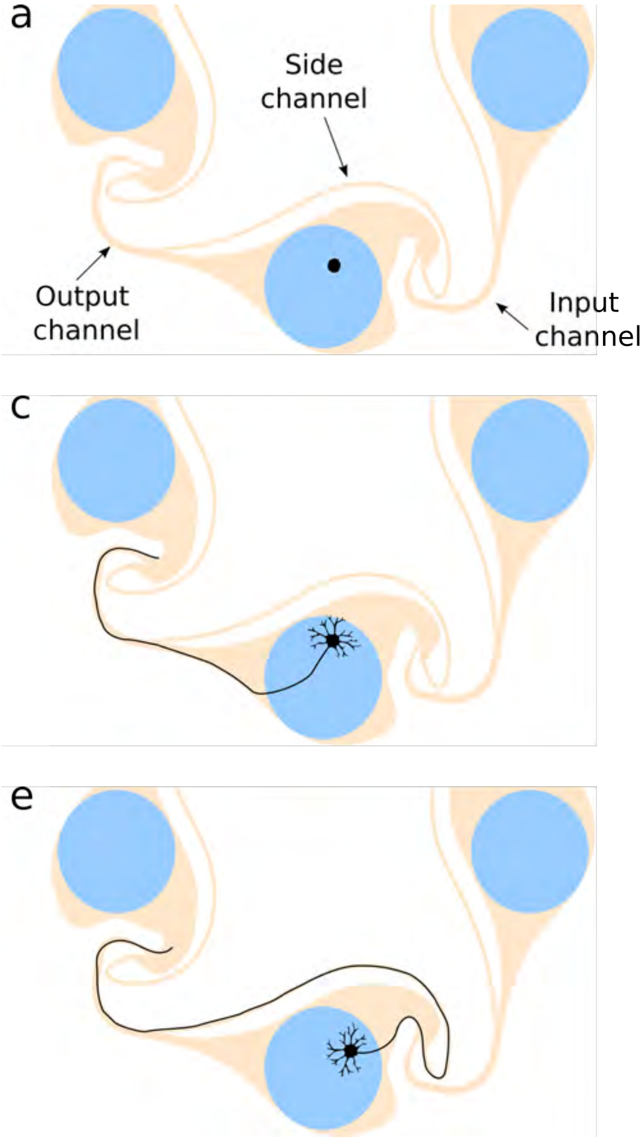

Figure S 16 Schematics of all the possible directions an axon can grow into after cell seeding. (a) Name of the channels used to describe possible axon outgrowth in the text (with regards to the node where the cell is seeded). Cases (b) to (e) show desirable, clockwise axonal growth direction. Case (f) is the undesirable, counter-clockwise axonal growth direction

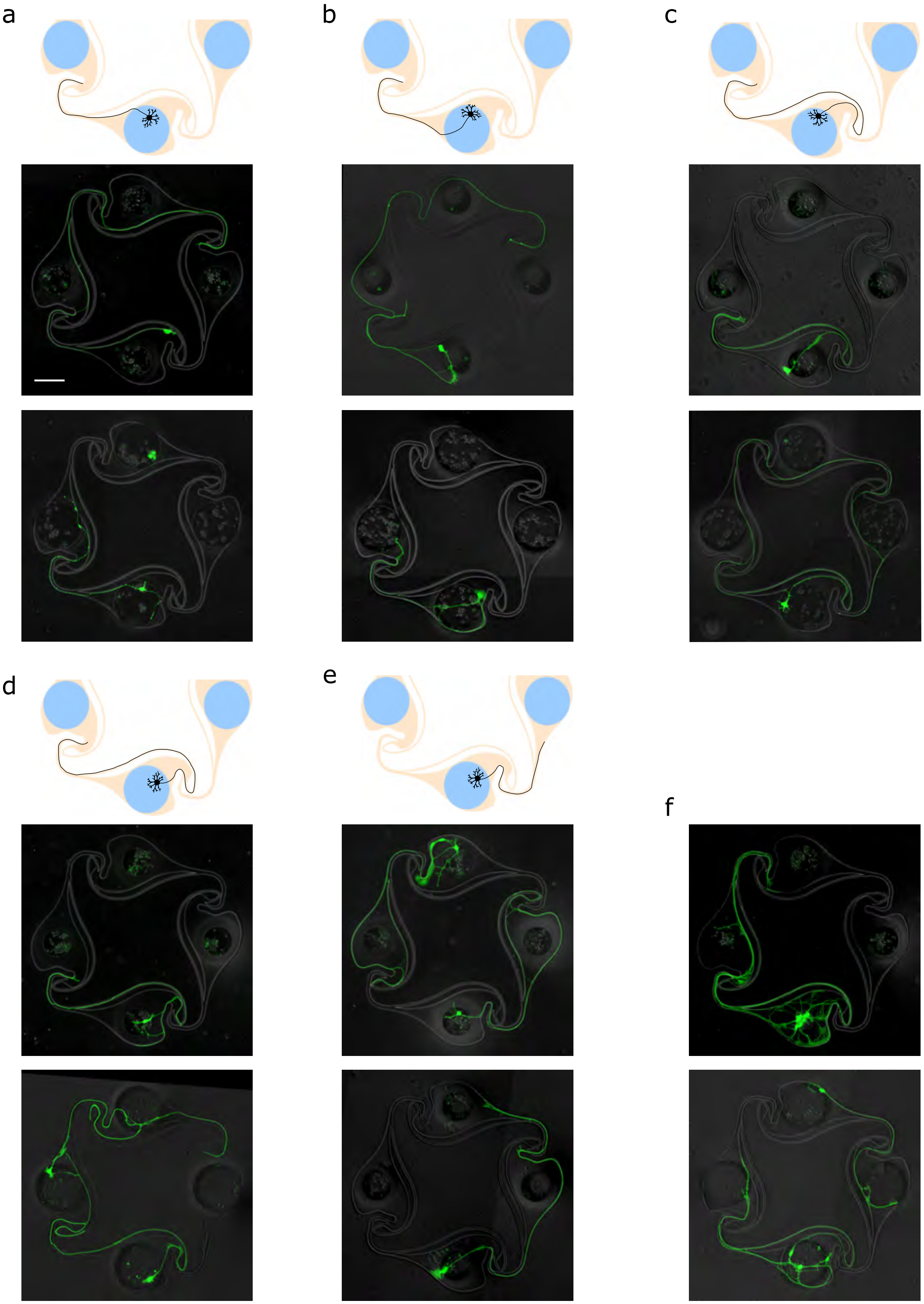

Figure S 17 Images of circuit with single nodes occupied by iNeurons, illustrating the five possible growth directions for axons in both 100 and 170 μm diameter nodes (a-e). (f) shows cases where several iNeurons survived in a well and their axons still followed the intended clockwise growth direction. Scale bar: 100 μm.

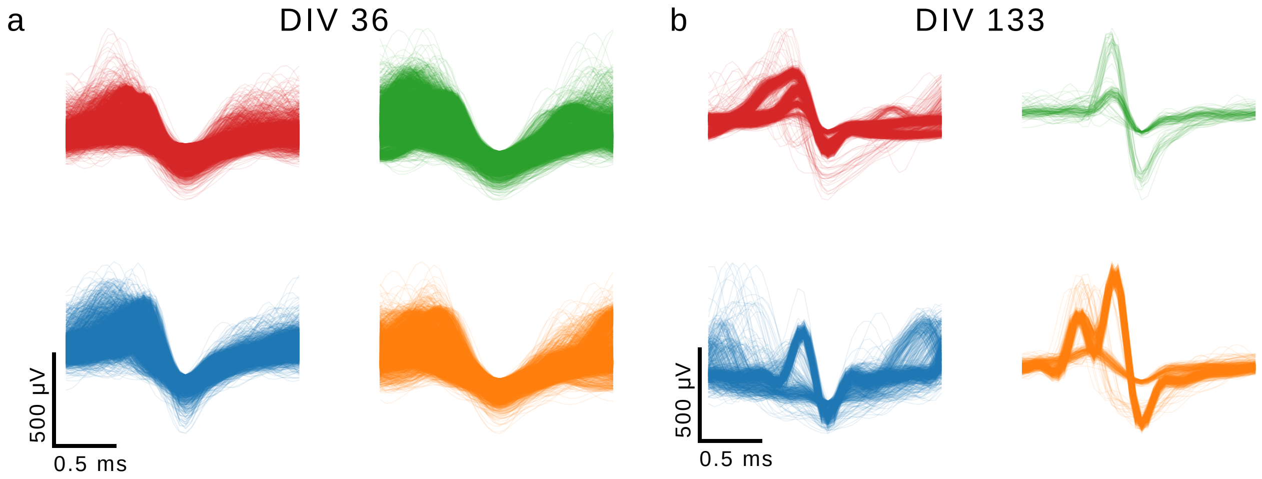

Figure S 18 Overlay of the waveforms of the action potentials detected in a 5 min recording of spontaneous electrical activity for the four electrodes shown on Fig. 6 at DIV 36 (a) and 133 (b)

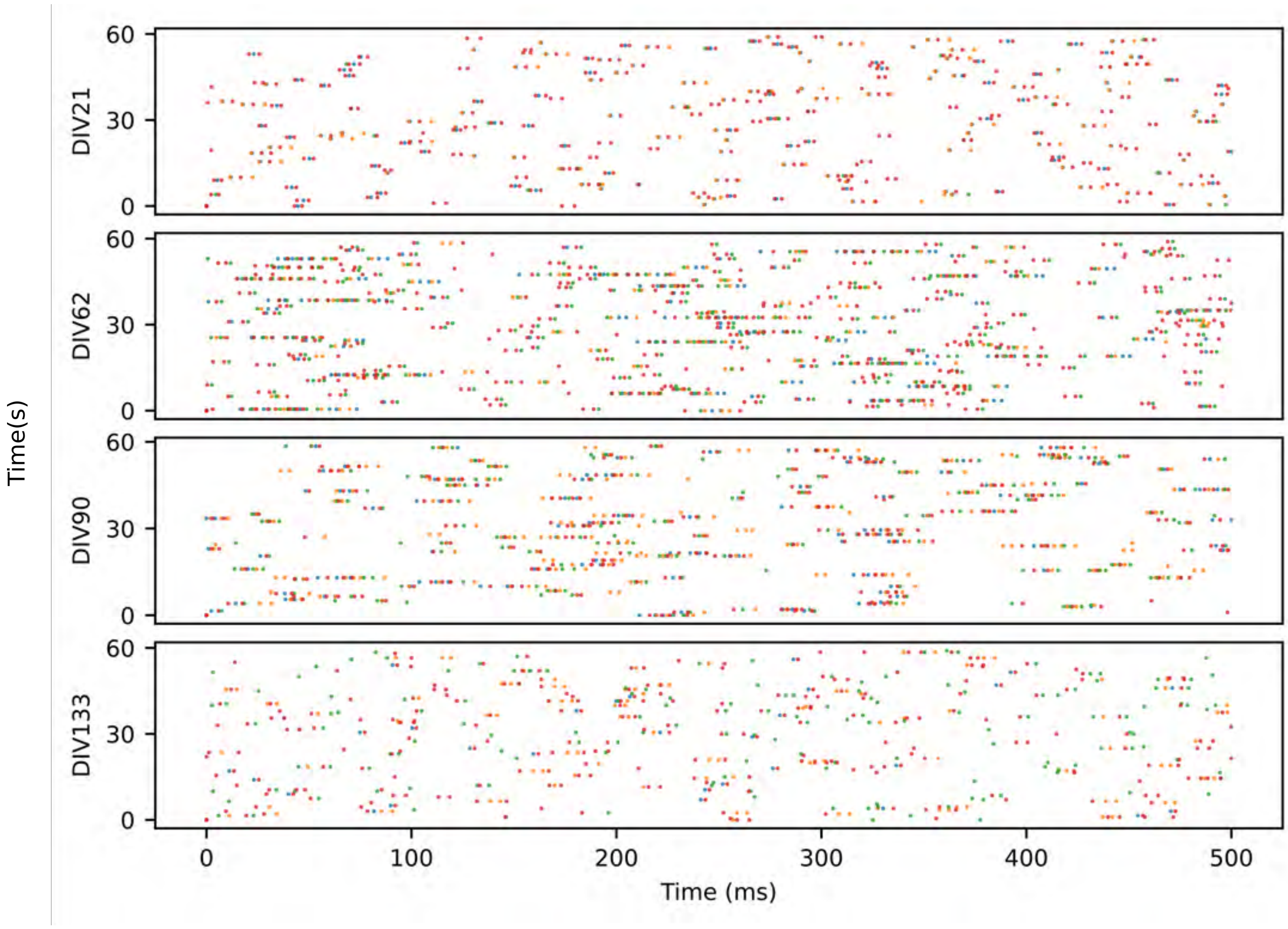

Figure S 19 Overlaid raster plots of the four electrodes of a the circuit shown in Fig. 6, with each electrode color-coded (top left: red; top right: green; bottom right: yellow; bottom left: blue)

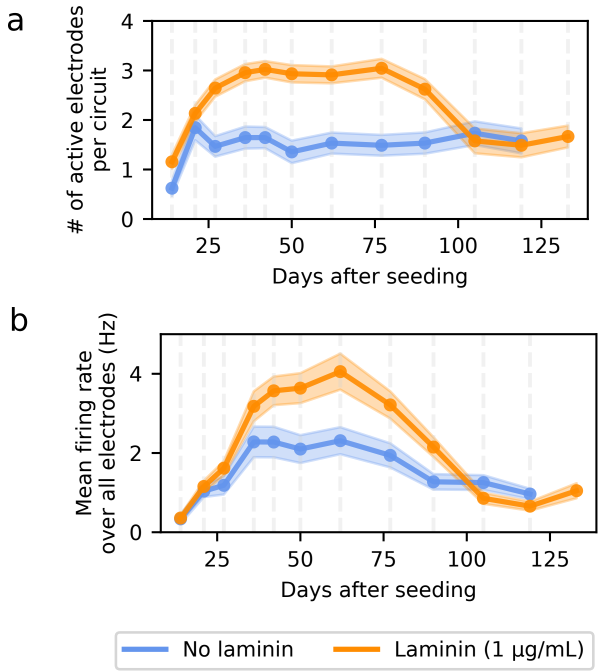

Figure S 20 Spontaneous electrical activity of iNeurons circuits cultured in regular medium (blue) and in medium supplemented with 1 µg/mL of laminin (orange). Data were recorded from 3 MEAs for each condition, at DIV 0, 14, 21, 27, 36, 42, 50, 62, 77, 90, 105, 119, and 133 ("laminin" samples only on DIV 133). (a) Average number of active electrodes per circuit (b) Mean firing rate across all electrodes (inactive and active ones). For both (a) and (b), the shaded area represents the SEM and N = 180 electrodes for each point.

a

b

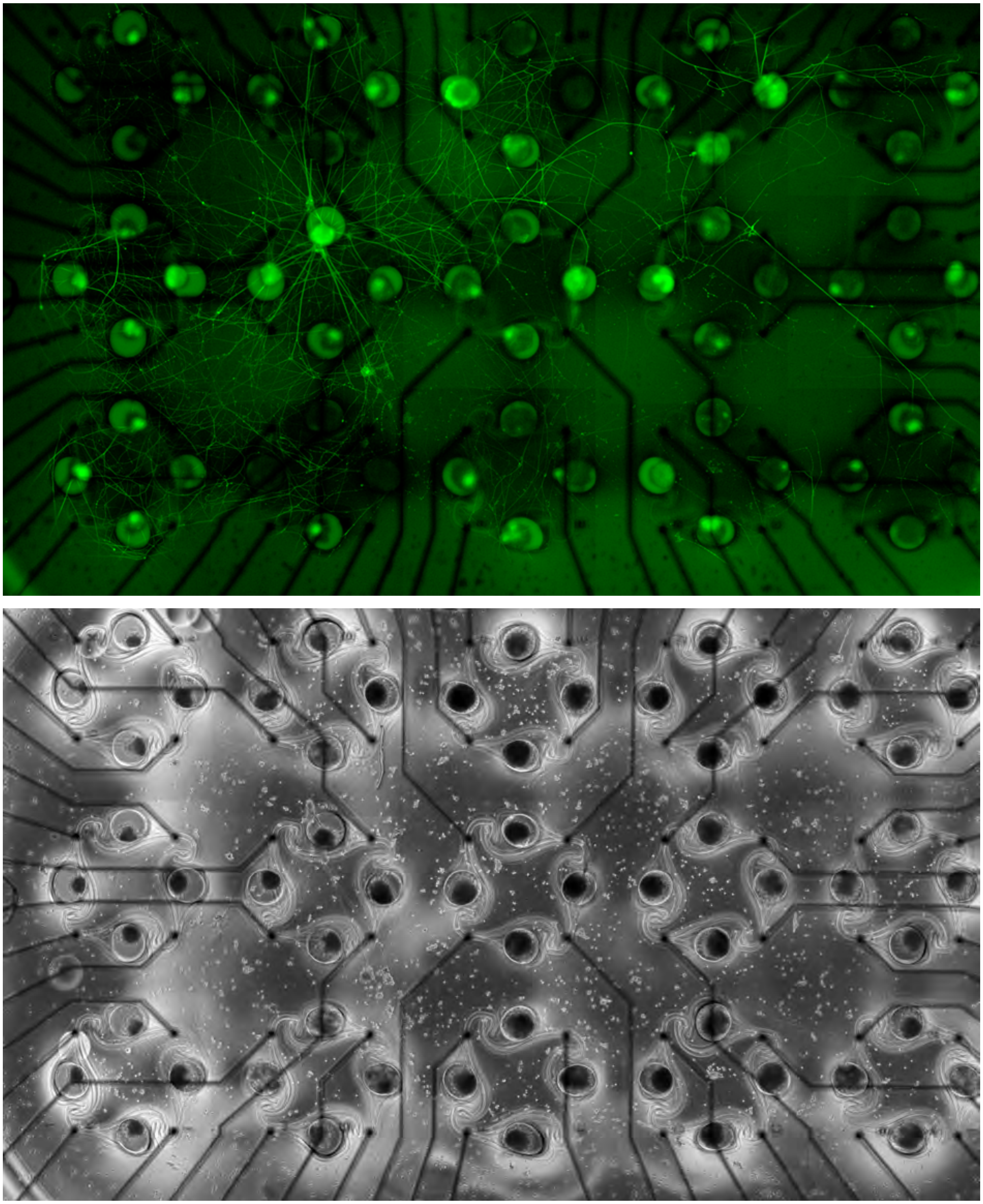

Figure S 21 Stitched image showing axons growing on top of a PDMS microstructure on a MEA after 91 DIV. (a) CMFDA stain. (b) Phase-contrast image.

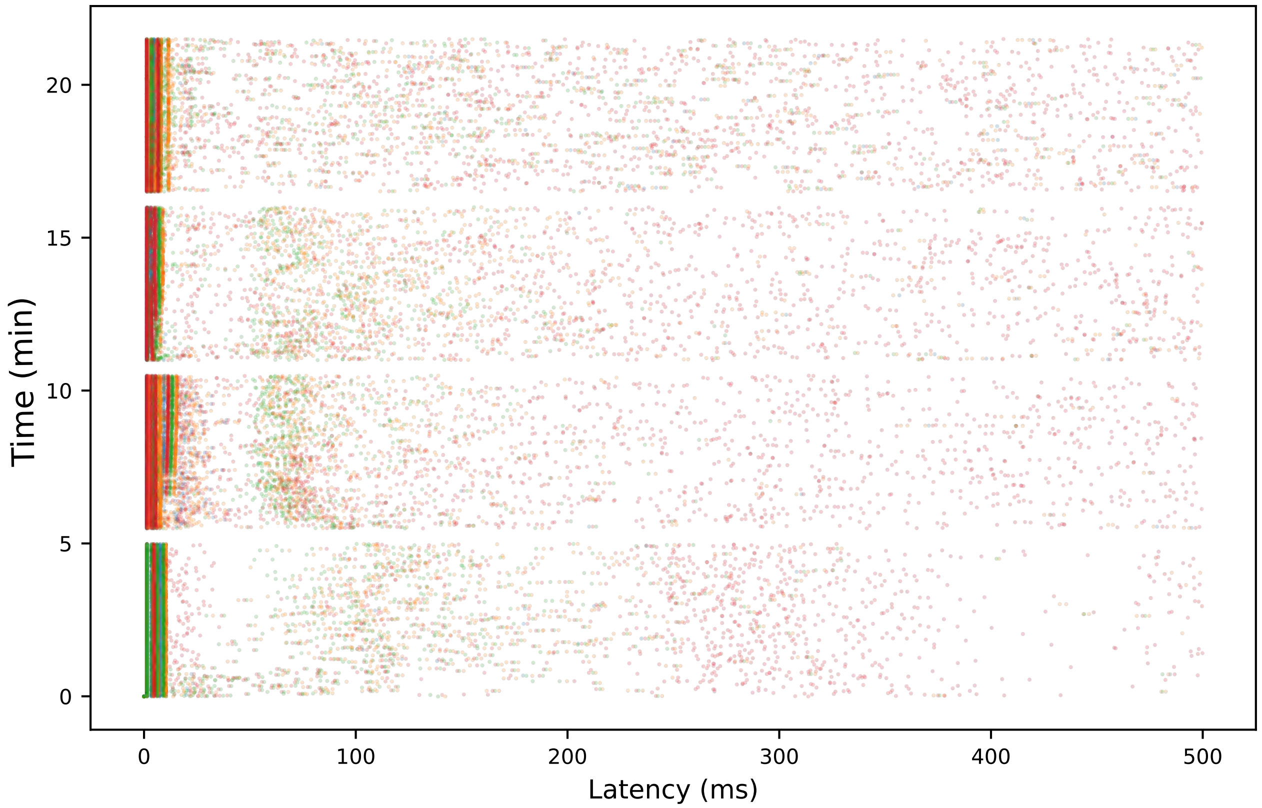

Figure S 22 Representative full electrical activity elicited at DIV 21 by a sequential 5 min stimulation of each of the electrodes of the circuit shown in Fig. 9d. A 2 Hz stimulus was sequentially applied to the top left (t = 0 to 5 min), top right (t = 5:30 to 10:30 min), bottom right (t = 11 to 16 min) and bottom left (t = 16:30 to 21:30 min) electrodes of the circuit.
